# Supplementary material for: Singularity response reveals entrainment properties in mammalian circadian clock
Source: Nat Commun. 2023 May 17;14:2819. doi: 10.1038/s41467-023-38392-x (PMC10192398; doi:10.1038/s41467-023-38392-x)
Supplement: Supplementary file 1 — Supplementary Information [file 41467_2023_38392_MOESM1_ESM.pdf]

Singularity response reveals entrainment properties in mammalian circadian clock

Supplementary Notes 1-3

Supplementary Figs. 1-14

Supplementary Note 1. Mathematical model for estimation of amplitude dependency of PRC at cellular and population rhythms.

A: Model for single-cellular PRC

Suppose that the circadian clock at the single-cell level is represented by a circular limit cycle with amplitude A. That is, the state of the cellular rhythm is represented by $(A\cos\theta, A\sin\theta)$ with amplitude A and phase θ. Here, we consider that the state of the circadian clock at the single-cell level moves by a distance of F in a direction ϕ, regardless of the phase and amplitude at the stimulation (Fig. 1). The state of the circadian rhythm after stimulation is expressed as $(A\cos\theta + F\cos\phi, A\sin\theta + F\sin\phi)$. Therefore, the difference in amplitude and phase before and after stimulation is expressed as follows,

$$dA = \sqrt{(A\cos\theta + F\cos\phi)^2 + (A\sin\theta + F\sin\phi)^2} - A, \quad \text{(S1)}$$

$$d\theta = \tan^{-1}\left(\frac{F\sin(\phi - \theta)}{A + F\cos(\phi - \theta)}\right). \quad \text{(S2)}$$

In Fig. 1b, $F = 0.1$ and $\phi = 0.75(\mathrm{rad}/2\pi)$.

B: Model for population PRC

We assumed that the rhythm of individual cells in the population is a sinusoidal rhythm with amplitude 1. In this case, the phase and amplitude of the rhythm at the population level are expressed as follows when the phase distribution of the cell population is $f(\theta)$,

$$Re^{i\Theta} = \int_0^{2\pi} f(x)\, e^{ix} dx. \quad \text{(S3)}$$

Here, R is the synchronization rate, which reflects amplitude of population rhythm, and Θ is the mean of phase, which is the phase of population rhythm. In this study, we considered a phase distribution that can be separated into two populations, a synchronized population and a desynchronized population, which was proposed in a previous study (*12*) as follows,

$$f(\theta) = R\delta(\theta - \Theta) + (1 - R)/2\pi. \quad \text{(S4)}$$

Here, $\delta(\theta)$ is the Dirac's delta function. We assume that phase of each cell response to the stimulation depending on the cellular-level PRC $g(x)$. In this case, R_2 and Θ_2, the amplitude and phase of population after the stimulation, are described as follows,

$$R_2 e^{i\Theta_2} = \int_0^{2\pi} f(x) e^{i\{x+g(x)\}} dx = Re^{i\{\Theta + g(\Theta)\}} + (1 - R)R' e^{i\Theta'}. \quad \text{(S5)}$$

Here, R' and Θ' are constants determined by $g(x)$ as

$$R' e^{i\Theta'} = \frac{1}{2\pi}\int_0^{2\pi} e^{i\{x+g(x)\}} dx. \quad \text{(S6)}$$

Consequently, amplitude response and phase response at the population level is described as,

$$dR = \sqrt{\left(R\sin(\Theta + g(\Theta)) + (1-R)R'\sin\Theta'\right)^2 + \left(R\cos(\Theta + g(\Theta)) + (1-R)R'\cos\Theta'\right)^2} - R, \qquad \text{(S7)}$$

$$d\theta = \tan^{-1}\left(\frac{R\sin\left(g(\Theta)\right) + (1-R)R'\sin(\Theta' - \theta)}{R\cos\left(g(\Theta)\right) + (1-R)R'\cos(\Theta' - \theta)}\right). \qquad \text{(S8)}$$

In Fig. 1b, $g(x) = 0.1\sin(x - \pi/2)$.

<u>C: Model for population PRC with cellular amplitude changes</u>

When we consider that the phase and amplitude are distributed according to $f_\theta(\theta)$ and $f_A(A)$, respectively, $f_\theta(\theta)$ and $f_A(A)$ satisfy the following equations,

$$\int_0^\infty \int_0^{2\pi} f_\theta(\theta) f_A(A)\, d\theta\, dA = 1, \qquad \text{(S9)}$$

$$\int_0^\infty \int_0^{2\pi} f_\theta(\theta) f_A(A) A e^{i\theta}\, d\theta\, dA = \bar{A} e^{i\bar{\Theta}}. \qquad \text{(S10)}$$

Here, $\bar{A}$ and $\bar{\Theta}$ are the amplitude and phase of population rhythm, respectively.

If we consider that, as in the model for single-cell PRC, the perturbation changes the state of each oscillator by $Fe^{i\phi}$, the state of population rhythm is

$$\int_0^\infty \int_0^{2\pi} f_\theta(\theta) f_A(A)\left(Ae^{i\theta} + Fe^{i\phi}\right) d\theta\, dA$$

$$= \int_0^\infty \int_0^{2\pi} f_\theta(\theta) f_A(A) A e^{i\theta}\, d\theta\, dA + Fe^{i\phi} \int_0^\infty \int_0^{2\pi} f_\theta(\theta) f_A(A)\, d\theta\, dA$$

$$= \bar{A} e^{i\bar{\Theta}} + Fe^{i\phi} \qquad \text{(S11)}$$

Therefore, when A and θ is replaced with $\bar{A}$ and $\bar{\Theta}$ in Eqs. (S1) and (S2), respectively, the results are same as the single-cellular PRC.

Supplementary Note 2. Estimation of correction coefficient β.

Finding the proportional coefficient β between the SR amplitude and the PRC amplitude is necessary for PRC estimation, as shown in Fig. 2g and Fig. 4c. However, since we have not measured PRCs of MEFs corresponding to the SRs, β cannot be obtained directly. On the other hand, when the circadian rhythm shows the strongest response, the amplitude of PRC is $R' \cong 1$. Hence, putting the SR amplitude for a sufficiently strong stimulus to $\hat{R}'_{\mathrm{max}}$, β is estimated as $\beta \cong 1/\hat{R}'_{\mathrm{max}}$. In NIH3T3 cells, the maximum value of $\hat{R}'_{\mathrm{max}}$ is 0.39 (R' = 0.92 at this time), thus β is estimated as 1/0.39 ≅ 2.56. The error of the estimated β is about 12% compared to that obtained in Fig. 2g. We can also estimate $\hat{R}'_{\mathrm{max}}$ ≅0.75 in MEFs, since the SR amplitude is saturated in the highest concentration (Fig. 5e). Consequently, β for *PER2*::*LUC* MEF is estimated as $\beta \cong 1/0.75 \cong 1.33$.

Supplementary Note 3. Analytical solution for $g_{model}(\phi)$.

Putting $\psi = \phi - b$ in Eq. (9) under the stimulation (i.e. $E(t) = 1$), the following equation is obtained:

$$\frac{d\psi}{dt} = \omega + a \sin\psi. \tag{S12}$$

Since Eq. (S12) does not include b, its solution ψ is independent of b.

Dividing both sides of Eq. (S12) by the right-hand side yields

$$\frac{1}{\omega + a\sin\psi}\frac{d\psi}{dt} = 1. \tag{S13}$$

Integrating both sides of Eq. (S13) in $[0, \Delta t]$, we obtain

$$\int_{\psi(0)}^{\psi(\Delta t)} \frac{1}{\omega + a\sin\psi} d\psi = \Delta t. \tag{S14}$$

Putting $s = \tan\frac{\psi}{2}$, the variables are transformed as

$$\sin\psi = \frac{2s}{1+s^2}, \tag{S15}$$

$$d\psi = \frac{2}{1+s^2} ds, \tag{S16}$$

while the integration range is transformed as $\psi: \psi(0) \to \psi(\Delta t)$, $s: \tan\frac{\psi(0)}{2} \to \tan\frac{\psi(\Delta t)}{2}$.

Hence, the left-hand side of Eq. (S14) is described as follows:

$$\int_{\psi(0)}^{\psi(\Delta t)} \frac{1}{\omega + a\sin\psi} d\psi = \int_{\tan\frac{\psi(0)}{2}}^{\tan\frac{\psi(\Delta t)}{2}} \frac{1}{\omega + a\frac{2s}{1+s^2}} \frac{2}{1+s^2} ds$$

$$= \int_{\tan\frac{\psi(0)}{2}}^{\tan\frac{\psi(\Delta t)}{2}} \frac{2}{\omega(1+s^2)+2as} ds\,. \tag{S17}$$

Thus,

$$\int_{\tan\frac{\psi(0)}{2}}^{\tan\frac{\psi(\Delta t)}{2}} \frac{2}{\omega(1+s^2)+2as} ds \;= \Delta t. \tag{S18}$$

Depending on the relationship between ω and a, the solution of $\psi(\Delta t)$ is obtained as follows.

If $a < \omega$,

$$\psi(\Delta t) = 2\tan^{-1}\left\{\frac{\sqrt{\omega^2-a^2}}{\omega}\tan\left(\frac{\sqrt{\omega^2-a^2}}{2}\Delta t + \tan^{-1}\left(\frac{\omega}{\sqrt{\omega^2-a^2}}\left(\tan\frac{\psi(0)}{2}+\frac{a}{\omega}\right)\right)\right)-\frac{a}{\omega}\right\}. \tag{S19}$$

If $a > \omega$,

$$\psi(\Delta t) = 2\tan^{-1}\left\{\frac{\frac{a+\sqrt{a^2-\omega^2}}{a-\sqrt{a^2-\omega^2}}e^{\sqrt{a^2-\omega^2}\Delta t}-\frac{\omega\tan\frac{\psi(0)}{2}+a+\sqrt{a^2-\omega^2}}{\omega\tan\frac{\psi(0)}{2}+a-\sqrt{a^2-\omega^2}}}{\frac{\omega}{a-\sqrt{a^2-\omega^2}}\left(\frac{\omega\tan\frac{\psi(0)}{2}+a+\sqrt{a^2-\omega^2}}{\omega\tan\frac{\psi(0)}{2}+a-\sqrt{a^2-\omega^2}}-e^{\sqrt{a^2-\omega^2}\Delta t}\right)}\right\} \tag{S20}$$

If $a = \omega$,

$$\psi(\Delta t) = 2\tan^{-1}\left\{\frac{\tan\frac{\psi(0)}{2}+\left(\tan\frac{\psi(0)}{2}+1\right)\omega\Delta t}{1-\left(\tan\frac{\psi(0)}{2}+1\right)\omega\Delta t}\right\} \tag{S21}$$

With respect to ψ, the phase response function, $g_\psi(\psi(0))$, to stimulus duration of Δt is defined as follows:

$$g_\psi(\psi(0)) = \psi(\Delta t) - \psi(0) - \omega\Delta t. \tag{S22}$$

$g_\psi(\psi)$ is independent of b because $\psi(\Delta t)$ is independent of b.

Since $\psi = \phi - b$ and $g_{model}(\phi(0)) = \phi(\Delta t) - \phi(0) - \omega\Delta t$,

$$\begin{aligned} g_{model}(\phi(0)) &= (\phi(\Delta t)-b)-(\phi(0)-b)-\omega\Delta t \\ &= \psi(\Delta t)-\psi(0)-\omega\Delta t \\ &= g_\psi(\psi(0)) \end{aligned} \tag{S23}$$

Using the phase response function $g_{model}(\phi(0))$, the singularity response quantities, R' and Θ', are given by the following equation:

$$R'e^{i\Theta'} = \frac{1}{2\pi}\int_0^{2\pi} e^{i(\phi + g_{model}(\phi) + \omega\Delta t)} d\phi \tag{S24}$$

Since $\phi = \psi + b$, $g_\phi(\phi) = g_\psi(\psi)$ and $d\phi = d\psi$,

$$R'e^{i\Theta'} = \frac{1}{2\pi}\int_{-b}^{2\pi - b} e^{i(\psi + b + g_\psi(\psi) + \omega\Delta t)} d\psi. \tag{S25}$$

Hence,

$$R'e^{i(\Theta' - b)} = \frac{1}{2\pi}\int_{-b}^{2\pi - b} e^{i(\psi + g_\psi(\psi) + \omega\Delta t)} d\psi. \tag{S26}$$

Because $g_\psi(\psi)$ is independent of b and the result of integration over one cycle is independent of the integration range ($[0,2\pi]$ or $[-b, 2\pi - b]$), the right-hand side of Eq. (S26) is independent of b and thus described as follows:

$$R'e^{i(\Theta' - b)} = H(a, \Delta t)e^{iI(a,\Delta t)}. \tag{S27}$$

Therefore, the following relationship is obtained:

$$R' = H(a, \Delta t), \tag{S28}$$

$$\Theta' = b + I(a, \Delta t). \tag{S29}$$

Supplementary Fig. 8 shows one-to-one correspondence between a and R' and between b and Θ'. Using these relationships, we can determine a and b such that R' and Θ' are consistent with the experimental values. Using the estimated a and b, the PRC $g_{model}(\phi)$ can be drawn using Eq. (S23).

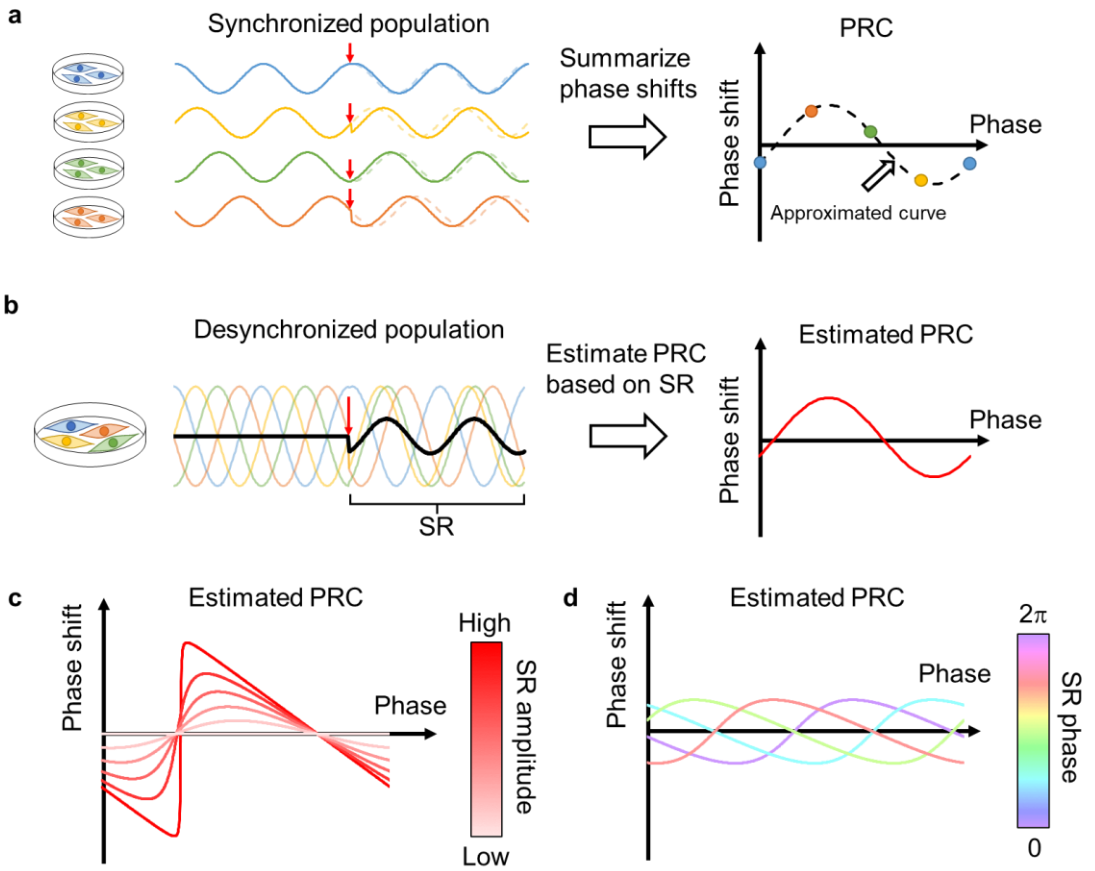

Supplementary Fig. 1 Relationship between PRC and SR. a Experimental scheme of the conventional PRC measurement method. The circadian phases of cells are synchronized before the measurement, and synchronized cell populations are stimulated at multiple different phases. Phase responses are obtained from each experiment, which are summarized as a PRC. **b** Experimental scheme of the SR method. The circadian phases of cells are desynchronized before the stimulation and the response of desynchronized population (i.e. singularity response, SR) is measured. The parameters of SR are then used to estimate the PRC. The colored lines and black line indicate the rhythms of individual cells and cell population, respectively. The red arrow indicates a stimulus. **c** Relationship between amplitude of the PRC and the SR. The higher the amplitude of the PRC, the higher the amplitude of the SR. **d** Relationship between phase of the PRC and the SR. The phase of the SR shifts as much as the phase of the stable point of the PRC shifts.

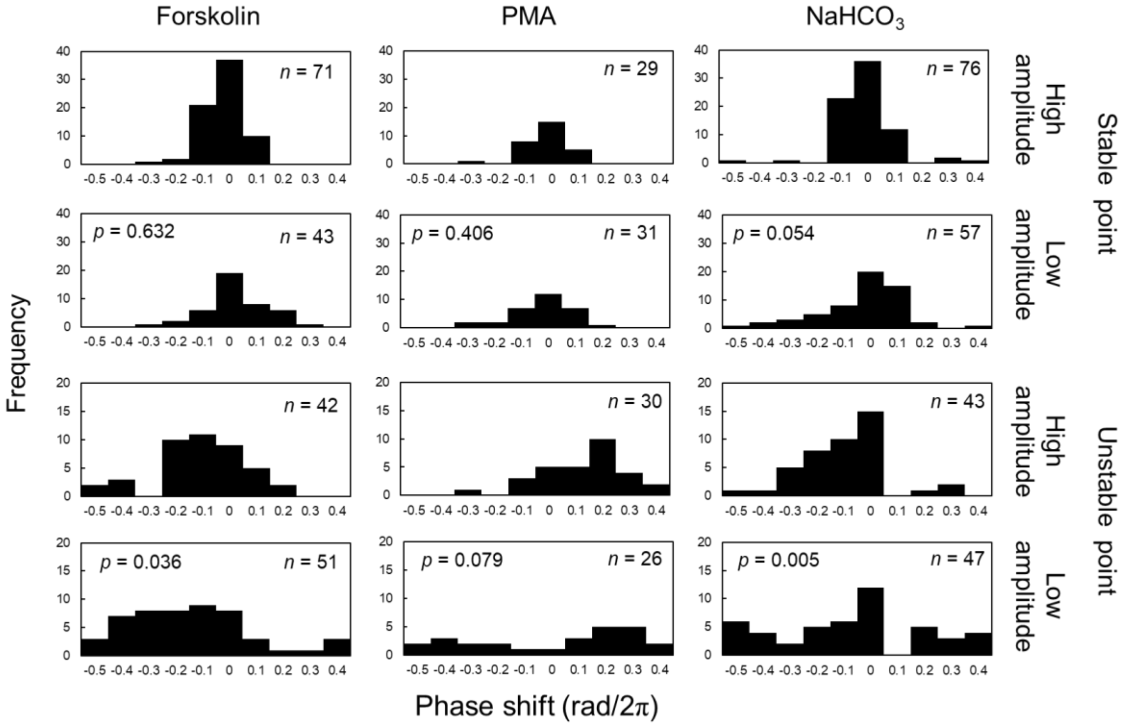

Supplementary Fig. 2 Distribution of phase shift around the stable/unstable point in high or low amplitude state in single-cellular PRC. The phase around the stable points in PRC was defined as 0-0.2 (rad/2π) for forskolin, 0.5-0.7 (rad/2π) for PMA, and 0.3-0.5 (rad/2π) for $NaHCO_3$. The phase around the unstable points was defined as 0.5-0.7 (rad/2π) for forskolin, 0-0.1 (rad/2π) for PMA, and 0.9-1.0 (rad/2π) for $NaHCO_3$. High amplitude was defined as $A > 0.6$ and low amplitude as $A < 0.4$. p-values represent the results of comparison between high and low amplitude states (Mardia-Watson-Wheeler test). The cell numbers were presented in each figure.

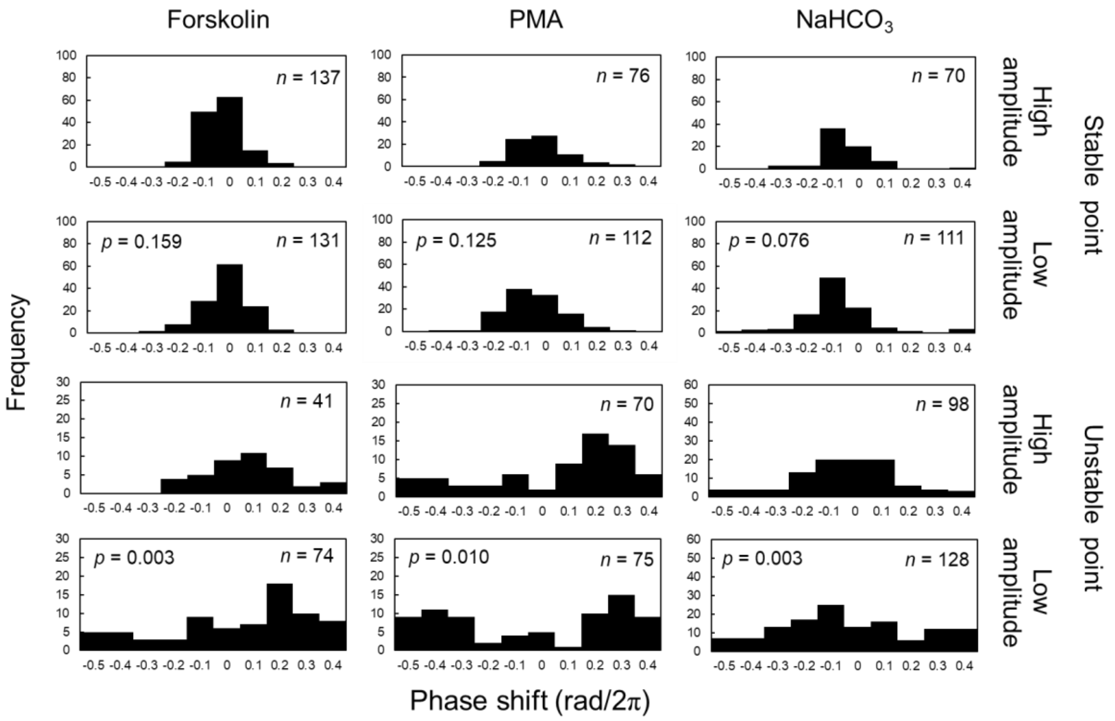

Supplementary Fig. 3 Distribution of phase shift around the stable/unstable point in high/low amplitude state in population PRC. The phase around the stable points was defined as 0.1-0.2 (rad/2π) for forskolin and 0.5-0.6 (rad/2π) for PMA and $NaHCO_3$. The phase around the unstable points was defined as 0.7-0.8 (rad/2π) for forskolin, 0.9-1.0 (rad/2π) for PMA, and 0.0-0.1 (rad/2π) for $NaHCO_3$. High amplitude was defined as $R > 0.8$ and low amplitude as $R < 0.1$. *p*-values represent the results of comparison between high and low amplitude state (Mardia-Watson-Wheeler test). The sample numbers were presented in each figure (each sample was obtained from five randomly selected cells).

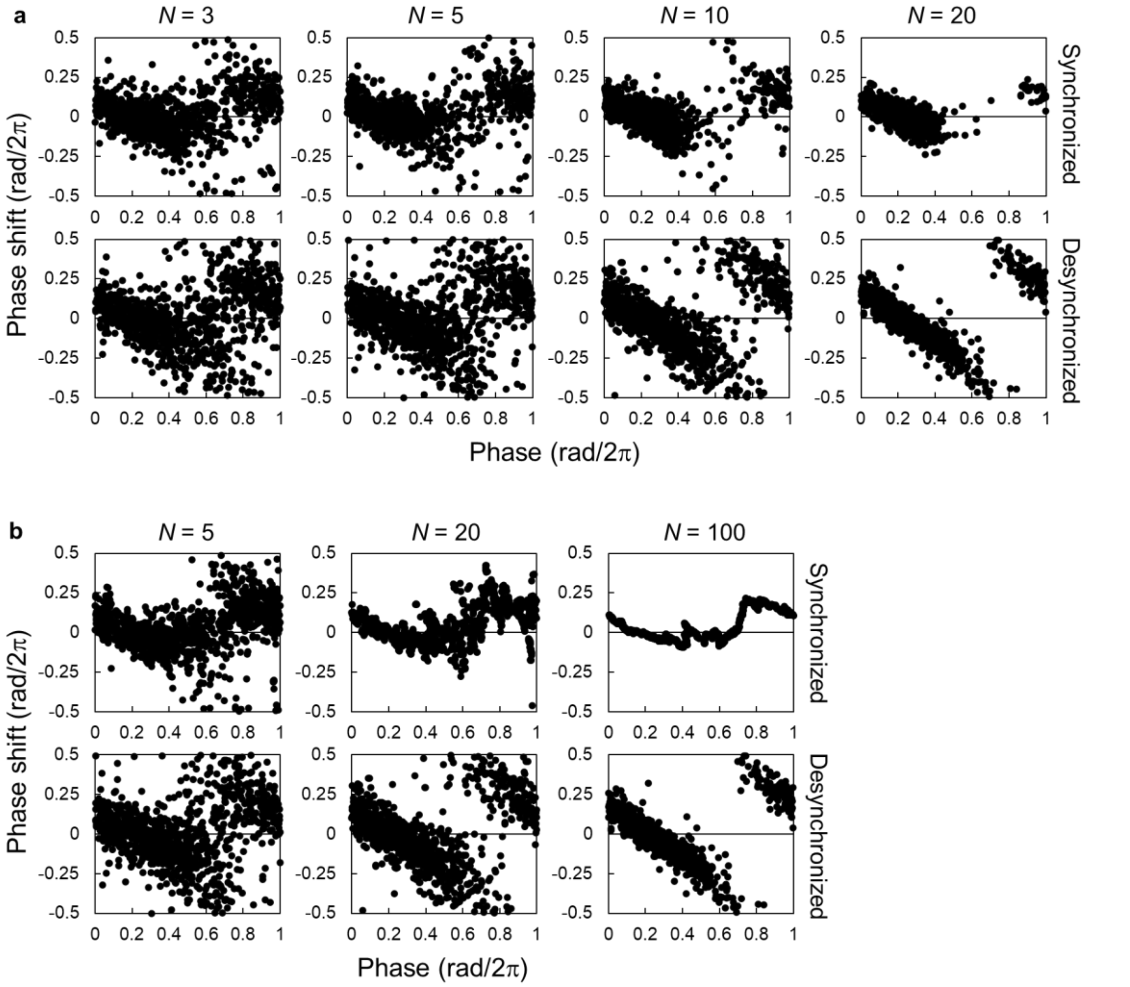

Supplementary Fig. 4 PRCs for forskolin in the cell populations. a PRCs for forskolin in the cell populations of various sizes. Each population is composed of indicated numbers of cells, which were randomly selected. Concentrations of forskolin was 0.5 μM. Amplitude (synchronization index) of $R > 0.8$ was defined as synchronized, while $R < 0.1$ was defined as desynchronized. **b** PRCs for forskolin in the cell populations of various sizes. The synchronized population was composed of the indicated numbers of cells with similar phase, while cells in the desynchronized population were randomly selected as in Fig. 1e and Supplementary Fig. 5a. The PRCs of the desynchronized population ($N = 5$ and 20) are the same as Supplementary Fig. 5a.

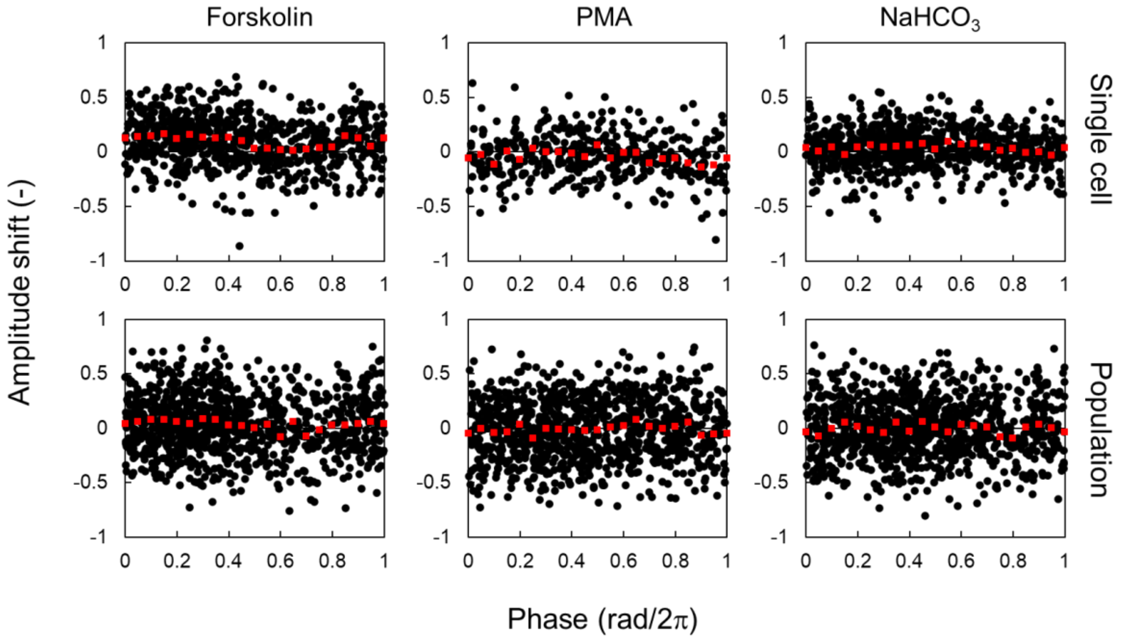

Supplementary Fig. 5 Amplitude response curves at single-cell and population levels. Single-cell amplitude response curve represents amplitude responses. Population amplitude response curve represents changes in synchronization rate (amplitude of population rhythms) (n = 1000). Red dots represent means of amplitude responses at 0.05 (rad/2π) intervals.

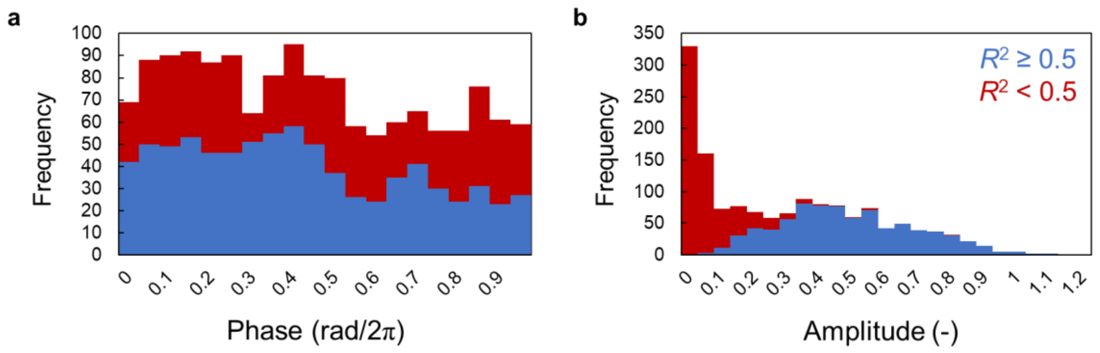

Supplementary Fig. 6 Phase distribution (a) and amplitude distribution (b) at the beginning of measurement. Each data point represents the distribution of phase of each cell at the beginning of measurement (a) and amplitude of each cellular rhythm (b) which was calculated using the recording data from 24 to 96 h in the untreated condition. R^2 is coefficient of determination of fitting curves. This indicated that the singularity state is a result of desynchronization in cultured cells rather than a decrease in the rhythm amplitude in a single cell.

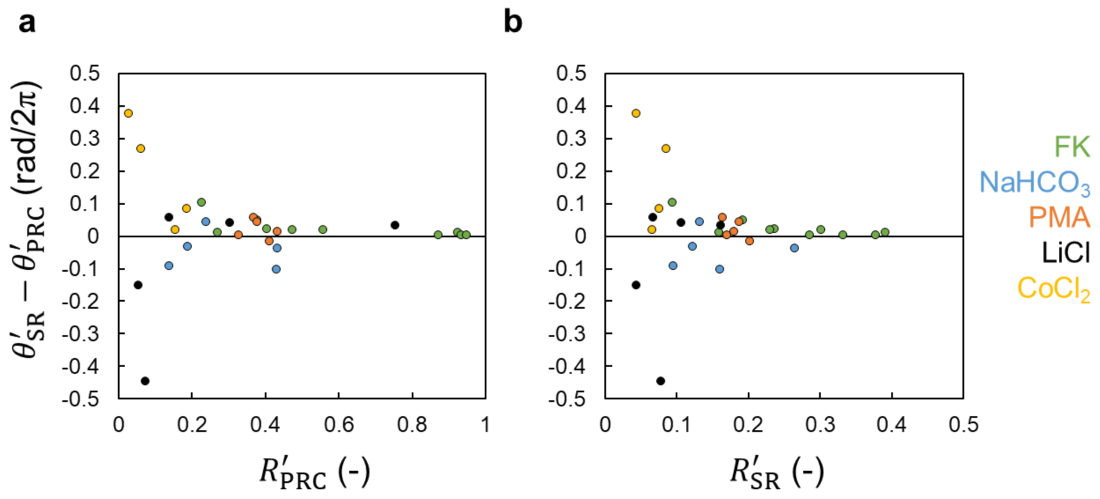

Supplementary Fig. 7 Relationship of error of estimated phase with strength of stimulation. a Error between SR phase and PRC phase and amplitude of PRC were plotted. **b** Error between SR phase and PRC phase and amplitude of SR were plotted.

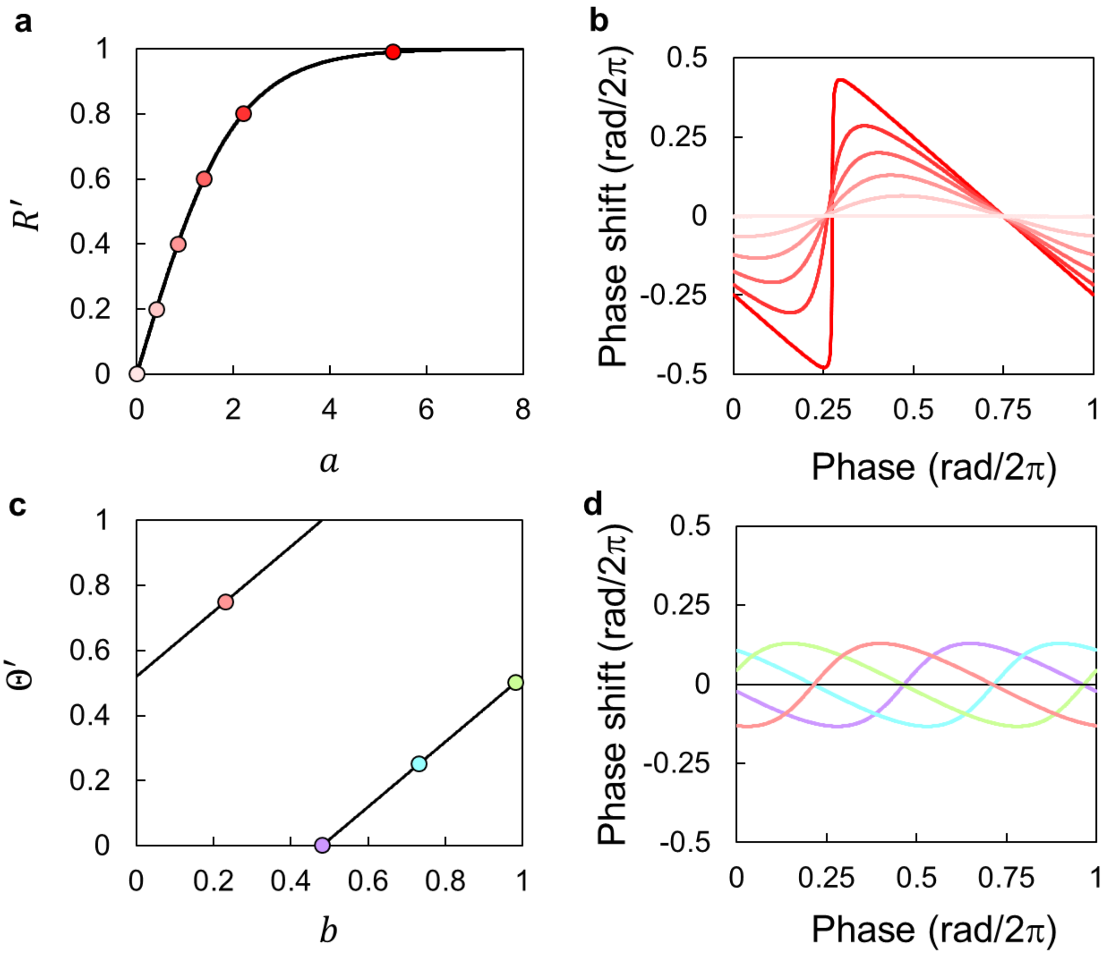

Supplementary Fig. 8 Models of relationship between the parameters of SR and measured PRC. a Relationship between the parameter *a*, representing the strength of the stimulation, and SR amplitude R' at duration of stimulus $\Delta t = 1$. Points at $R' = 0, 0.2, 0.4, 0.6, 0.8$ and 0.99 were plotted. **b** Change in PRC as a function of stimulus strength. Each PRC represents the PRC at $R' = 0, 0.2, 0.4, 0.6, 0.8$ or 0.99, $\Theta' = 0.75$ (rad/2/π) and $\Delta t = 1$. **c** Relationship between the phase characteristic *b* of the stimulus and the phase of SR at $R' = 0.4$ and $\Delta t = 1$. Each point represents a point at $\Theta' = 0, 0.25, 0.5$ or 0.75 (rad/2/π). **d** Change in PRC as a function of phase characteristic. Each PRC represents the PRC at $\Theta' = 0, 0.25, 0.5$ or 0.75 (rad/2/π), $R' = 0.4$, and $\Delta t = 1$. These plots were obtained from Eqs. (S19-S22) and (S25).

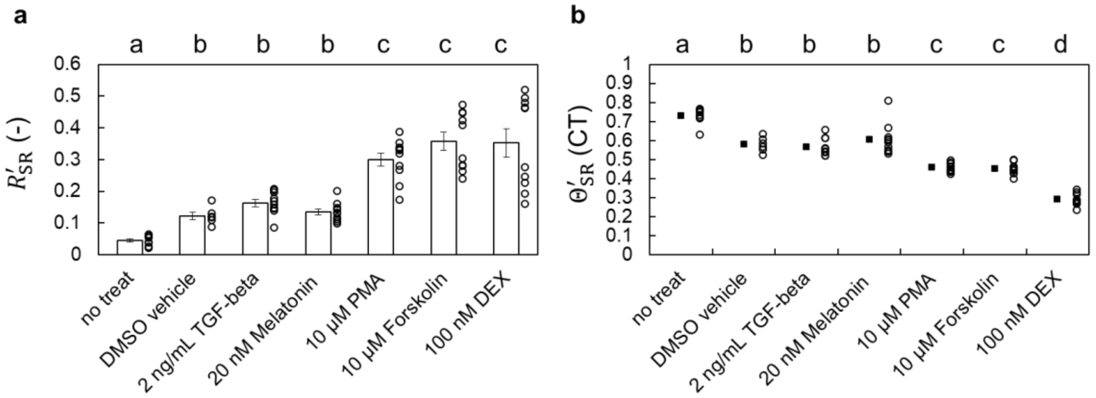

Supplementary Fig. 9 SR parameters in Rat-1 cells. a Amplitude of SR in Rat-1 cells (Mean ± SEM with individual plots, $n = 8$ biologically independent samples for no treat, $n = 6$ for DMSO, and $n = 10$ for the others). Conditions with different letters above the bars indicate significant differences with each other (Steel-Dwass test, $p < 0.05$). **b** Phase of SR in Rat-1 cells. Filled squares indicate the circular mean of phase and blank circles indicate individual data ($n = 8$ biologically independent samples for no treat, $n = 6$ for DMSO, and $n = 10$ for the others). Conditions with different letters indicate significant differences (Watson-Williams test with Bonferroni correction, $p < 0.05$).

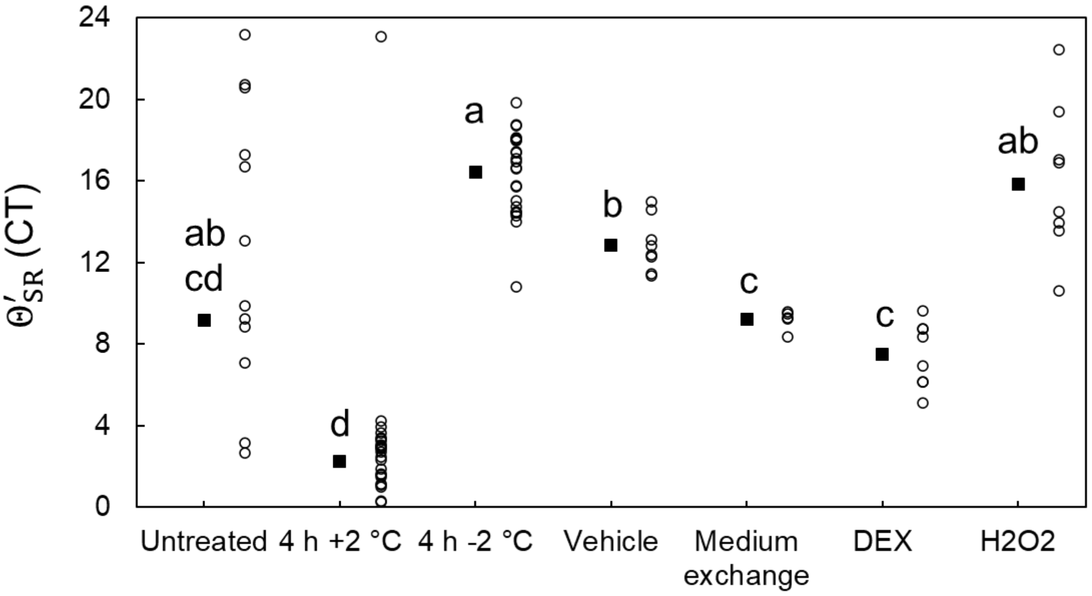

Supplementary Fig. 10 Phase of SR in MEFs. Filled squares indicate the circular mean of phase and blank circles indicate individual data (n = 12 biologically independent samples for untreated, 24 in 4 h ± 2 °C, n = 5 for medium exchange, and n = 8 for the others). Conditions with different letters indicate significant differences (Watson-Williams test with Bonferroni correction, $p < 0.05$).

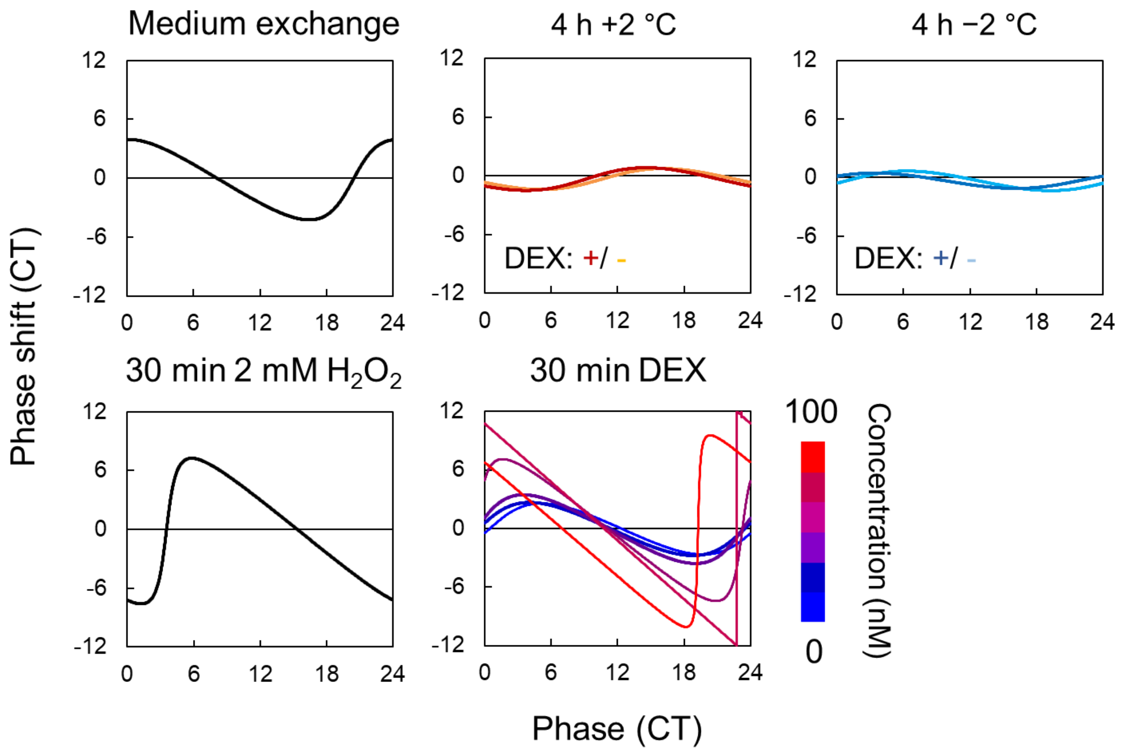

Supplementary Fig. 11 Estimated PRCs in MEFs. PRC for each stimulus was estimated using the SR. The concentrations of dexamethasone are 0, 0.16, 0.8, 4, 20 and 100 nM.

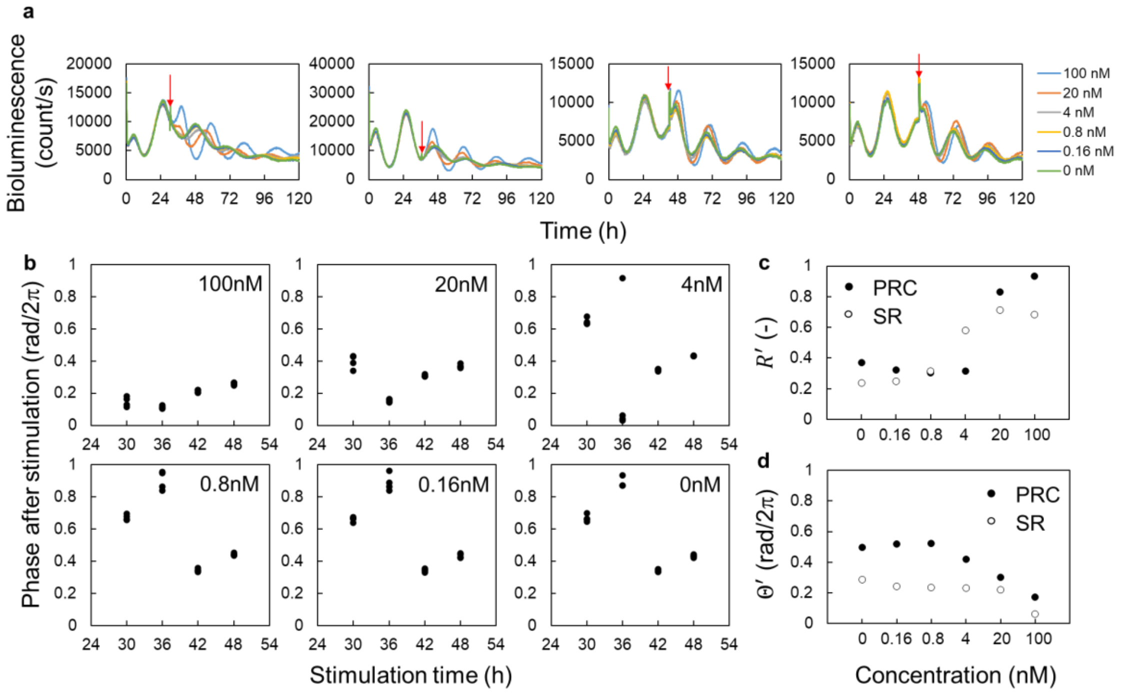

Supplementary Fig. 12 The phase responses for DEX stimulation at a series of concentrations. a The responses of *PER2*::*LUC* rhythm in MEF for DEX stimulation at a series of concentrations. Each stimulus was applied 30, 36, 42 and 48 h after the beginning of measurement. **b** Reset phases after DEX stimulation. **c and d** the synchronization index R' (**c**) and the average phase Θ' (**d**) after the DEX stimulation with each concentration. The black points are the R' and Θ' obtained from the PRC (**b**) and the white points are the amplitude and phase of SR (Fig. 5e).

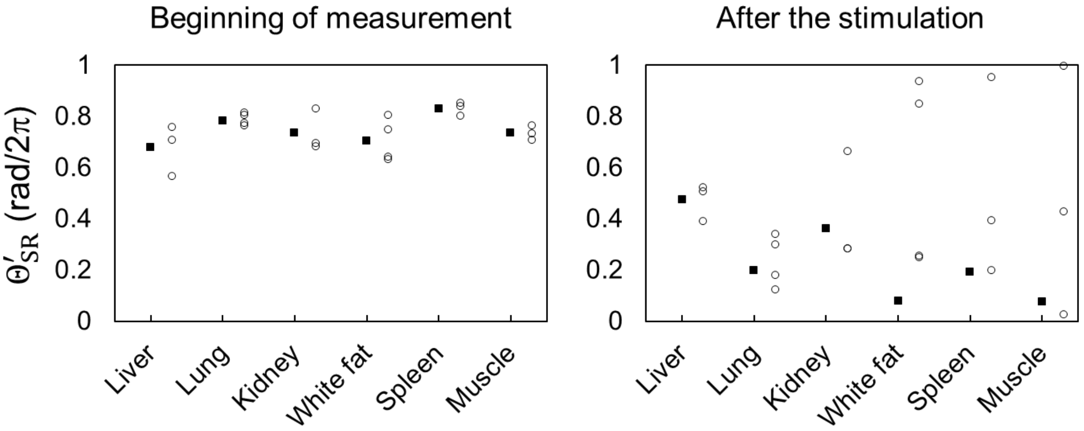

Supplementary Fig. 13 Phase responses of tissue slice cultures. We applied temperature stimuli (+2 °C for 4 hours) to slice cultures. Filled squares indicate means of phase and blank circles indicate individual data (n = 3 biologically independent samples for liver, kidney, spleen and muscle, n = 4 biologically independent samples for lung and white fat).

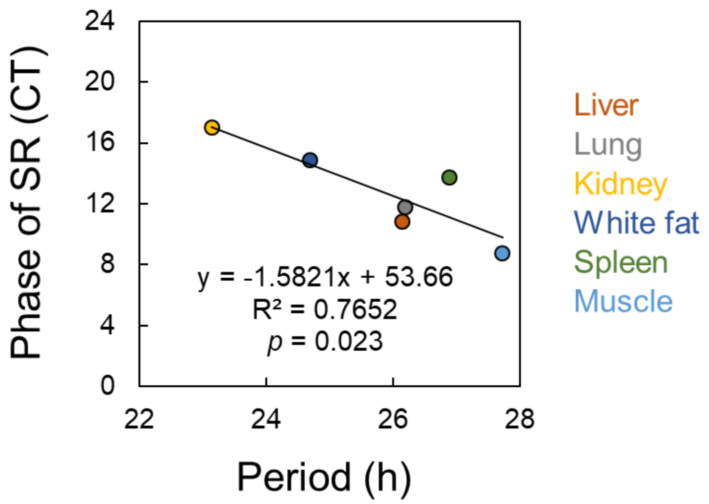

Supplementary Fig. 14 Phase responses of tissue slice cultures depend on the circadian period. The black line is the approximate straight line. R^2 represents the coefficient of determination. p represents the significance (two-tailed probability values) of a Pearson correlation coefficient.
