# Supplementary material for: Singularity response reveals entrainment properties in mammalian circadian clock
Source: Nat Commun. 2023 May 17;14:2819. doi: 10.1038/s41467-023-38392-x (PMC10192398; doi:10.1038/s41467-023-38392-x)
Supplement: Supplementary file 3 — Reporting Summary [file 41467_2023_38392_MOESM3_ESM.pdf]

nature portfolio

Corresponding author(s): Arisa Hirano

Last updated by author(s): Apr 5, 2023

Reporting Summary

Nature Portfolio wishes to improve the reproducibility of the work that we publish. This form provides structure for consistency and transparency in reporting. For further information on Nature Portfolio policies, see our Editorial Policies and the Editorial Policy Checklist.

Statistics

For all statistical analyses, confirm that the following items are present in the figure legend, table legend, main text, or Methods section.

n/a	Confirmed	
☐	☒	The exact sample size (*n*) for each experimental group/condition, given as a discrete number and unit of measurement
☐	☒	A statement on whether measurements were taken from distinct samples or whether the same sample was measured repeatedly
☐	☒	The statistical test(s) used AND whether they are one- or two-sided<br>*Only common tests should be described solely by name; describe more complex techniques in the Methods section.*
☒	☐	A description of all covariates tested
☐	☒	A description of any assumptions or corrections, such as tests of normality and adjustment for multiple comparisons
☐	☒	A full description of the statistical parameters including central tendency (e.g. means) or other basic estimates (e.g. regression coefficient) AND variation (e.g. standard deviation) or associated estimates of uncertainty (e.g. confidence intervals)
☐	☒	For null hypothesis testing, the test statistic (e.g. *F*, *t*, *r*) with confidence intervals, effect sizes, degrees of freedom and *P* value noted<br>*Give P values as exact values whenever suitable.*
☒	☐	For Bayesian analysis, information on the choice of priors and Markov chain Monte Carlo settings
☒	☐	For hierarchical and complex designs, identification of the appropriate level for tests and full reporting of outcomes
☒	☐	Estimates of effect sizes (e.g. Cohen's *d*, Pearson's *r*), indicating how they were calculated

Our web collection on statistics for biologists contains articles on many of the points above.

Software and code

Policy information about availability of computer code

Data collection	We used the software attached with the measurement device, Kronos HT (ATTO, Japan), for the bioluminescence measurements.
Data analysis	Python (version 3.9.5) with the library lmfit (version 1.0.3) was used for the calculation of phase and amplitude.<br>R (version 4.1.1) was used for the statical tests. In the multiple comparison test, we used the R package “multcomp” (version 1.4.18) for Tukey–Kramer test, and “NSM3” (version 1.16) for Steel–Dwass test. We also used the R package “circular” (version 0.4.93) for the assessment of circular data.<br>Procedures for other calculations are shown in the manuscript.

For manuscripts utilizing custom algorithms or software that are central to the research but not yet described in published literature, software must be made available to editors and reviewers. We strongly encourage code deposition in a community repository (e.g. GitHub). See the Nature Portfolio guidelines for submitting code & software for further information.

Data

Policy information about availability of data

All manuscripts must include a data availability statement. This statement should provide the following information, where applicable:

- Accession codes, unique identifiers, or web links for publicly available datasets
- A description of any restrictions on data availability
- For clinical datasets or third party data, please ensure that the statement adheres to our policy

All data needed to evaluate the conclusions of this study are presented in the manuscript and/or Supplementary Information. Source data are provided with this manuscript. Additional relevant data and materials may be requested from the authors.

Human research participants

Policy information about studies involving human research participants and Sex and Gender in Research.

Reporting on sex and gender	NA
Population characteristics	NA
Recruitment	NA
Ethics oversight	NA

Note that full information on the approval of the study protocol must also be provided in the manuscript.

Field-specific reporting

Please select the one below that is the best fit for your research. If you are not sure, read the appropriate sections before making your selection.

☒ Life sciences ☐ Behavioural & social sciences ☐ Ecological, evolutionary & environmental sciences

For a reference copy of the document with all sections, see nature.com/documents/nr-reporting-summary-flat.pdf

Life sciences study design

All studies must disclose on these points even when the disclosure is negative.

Sample size	No sample size calculation was performed, but the samples size is sufficient to show differences in responsiveness for each stimulus as shown in the manuscript.
Data exclusions	No data were excluded.
Replication	The experiments were performed at least two times to verify the reproducibility.
Randomization	Cell culture plates and animals were randomly allocated into the experimental groups.
Blinding	Blinding was not relevant to our study because all bioluminescence measurements and analysis after the treatments were automated.

Reporting for specific materials, systems and methods

We require information from authors about some types of materials, experimental systems and methods used in many studies. Here, indicate whether each material, system or method listed is relevant to your study. If you are not sure if a list item applies to your research, read the appropriate section before selecting a response.

Materials & experimental systems

n/a	Involved in the study
☒	☐ Antibodies
☐	☒ Eukaryotic cell lines
☒	☐ Palaeontology and archaeology
☐	☒ Animals and other organisms
☒	☐ Clinical data
☒	☐ Dual use research of concern

Methods

n/a	Involved in the study
☒	☐ ChIP-seq
☒	☐ Flow cytometry
☒	☐ MRI-based neuroimaging

Eukaryotic cell lines

Policy information about cell lines and Sex and Gender in Research

Cell line source(s)	PER2::LUC MEFs were established from PER2::LUC mice generated by Yoo et al. (Proc Natl Acad Sci U S A., 2004,101:5339-46).<br>We used Rat-1 Bmal1-luc cells generated in the previous study (Kon et al., Nat. Cell Biol., 2008, 12:1463-1469).
Authentication	The cell lines were not authenticated.
Mycoplasma contamination	The cell lines were not tested for mycoplasma contamination.
Commonly misidentified lines<br>(See ICLAC register)	No commonly misidentified cell lines were used.

Animals and other research organisms

Policy information about studies involving animals; ARRIVE guidelines recommended for reporting animal research, and Sex and Gender in Research

Laboratory animals	Male C57BL/6J PER2::LUC mice aged 31-39 weeks were used. PER2::LUC mice originate from mice generated by Yoo et al. (Proc Natl Acad Sci U S A., 2004,101(15):5339-46). Animals were maintained under a 12:12h light/dark cycle at 23.5 ± 2.0 °C and 50.0 ± 10.0 % humidity. Food and water were available ad libitum.
Wild animals	This study did not use wild animals.
Reporting on sex	The findings in the animal experiments can apply to only male mice; sex difference was not considered.
Field-collected samples	This study did not include samples collected from the field.
Ethics oversight	All experiments involving animals were approved by the Animal Experiment and Use Committee of the University of Tsukuba and were therefore in accordance with NIH guidelines.

Note that full information on the approval of the study protocol must also be provided in the manuscript.
